# Report of a Case of Gunshot Wound, in Which Five Shot Were Lodged in the Cavities of the Heart

**Published:** 1828-10

**Authors:** Leonard Randal

**Affiliations:** Purdy, Mc’Nary County, Tennessee


THE
### WESTERN JOURNAL
OF THE
### MEDICAL & PHYSICAL SCIENCES.

OCTOBER, 1828.

### ESSAYS AND CASES.

Art. I.—*Report of a case of Gunshot Wound of the Lungs and Heart, with the appearances after death .By* Leonard Randal, M. D. *of Tennessee.*

On the 5th of April 1828, a negro boy aged 15 years, was by accident, shot in the breast, with a common fowling piece loaded with shot. He stood six feet from the muzzle of the gun, and received the whole charge. All the shot, with the exception of two or three, entered near each other, within the area of a dollar, on the left side of the sternum, beginning about an inch and a half below its lower extremity. He fell to the ground immediately, on receiving the wound.

The accident happened at one o'clock. Dr. Hudspeth and myself were immediately sent for. I was gone to the country, but the Doctor repaired to the house without delay. His pulse was scarcely perceptible, and he breathed with great difficulty. The Doctor opened a vein, and took three or four ounces of blood, and gave him thirty-five drops of laudanum upon which his pulse rose a little; he also applied a poultice of bread and milk to the wound, to encourage the hæmorrhage which was not profuse. When I arrived two hours after the accident, I found his pulse very weak and irregular. We gave him stimulants, occasionally, which rendered it stronger and more steady; he expectorated blood freely. We gave him a dose of ol. ricini which his stomach rejected immediately; and while vomiting he discharged a large quantity of wind, suggesting to us that the external air found its way into the œsophagus, from that tube having been wounded; a supposition that was strengthened by his frequent subsequent eructations. We also gave him a dose of sulphate of magnesia, which like the oil was thrown up.

April 6. In the morning administered an opiate, and half an hour afterwards, ten grains of calomel with ten of rhubarb. In the evening we gave him half an ounce of oleum ricini, and about midnight an enema.

On the morning of the 7th, we administered an infusion of senna, about twelve another enema, and at three repeated the infusion. He had now become extremely restless, his pulse was very weak and intermittent, syncope came on, and he had the appearance of being in the jaws of death. We gave him camphorated spirit, he recovered, and his pulse became fuller, a degree of fever supervened, for which he took the sp. nitr. dulc. and it soon abated. About six he had an alvine discharge, the first he had experienced except what was brought away by the enemata. He immediately got much better, felt an inclination to eat, and took a cup of soup. In the course of the night he had two or three discharges more, and rested better than he had previously done, though he generally had a great disposition to sleep.

Morning of the 8th. Said he felt much better. About two o'clock in the afternoon, a fever rose and he became restless, complaining of great pain in the breast, and about the heart. There was some tension and swelling of the right hypochondrium and his feet and legs also swelled; he had two or three bloody alvine discharges, and was very restless through the night, although we kept him on a strict antiphlogistic regimen, and gave him an anodyne.

Next morning (9th) the wound began to slough, he was restless, pulse very weak, frequent and intermitting, extremities cold, he spit blood, and his respiration was difficult. We bathed him in warm water, and gave him wine; his pulse rose and his circulation became full and regular. The abdominal swelling abated, he felt much better, and took nourishment. At three o'clock in the afternoon fever rose, he became very restless, and complained of pain and heat about the heart. His pulse at the same time was weak and frequent, and his extremities cold, we bathed them in warm water, and gave him an anodyne, after which he became easy, and rested well all night.

The 10th. The wound continued sloughing. Towards evening his fever rose higher than usual, his skin became hot and dry, he was taken with a violent cough, which gave him great pain, we administered an expectorant mixture, and he rested well through the night.

On the 11th the wound had sloughed so considerably as to form a hole into the cavity of the thorax, two thirds of an inch in diameter. The greatest part of the bruised flesh, seemed indeed to have sloughed off, down to the cartilages of the ribs, one of which had been perforated by the shot. He felt some anxiety in the breast, especially about the heart, and made an effort to puke, when a considerable quantity of thin pus flowed from the wound, and afforded him relief; he took an anodyne and rested well that night.

On the 12th the discharge continued, the wound put on a healthy appearance, and began to granulate.

It continued gradually to heal, and in three or four weeks was completely cicatrized; the œdematous swelling of the lower extremities disappeared in a week or two after the last date, and although extremely emaciated, he was able to walk about and had many appearances of getting well. When in this promising condition, he relapsed, apparently from indulging himself too freely in a meal of strong diet. From this relapse he did not recover, a hectic fever supervened, and he died on the night of the 11th of June, sixty-seven days after the accident.

### Appearances after death.

Leave was obtained to open the body, to which I proceeded next morning, assisted by Dr. Hudspeth. In dissecting up the integuments, we found several shot lodged against the ribs. The membraneous covering of the ribs and cartilages, was in a state of inflammation, and bore slight marks of gangrene. On raising the sternum, I found part of the pericardium absorbed, and a part of it adhering to the surface of the heart, while near its base, it was morbidly attached to the pleura (mediastinum.) An adhesion of the left lobe of the lungs, to the pleura had also taken place; there was lodged in its cellular substance, at various depths, a number of shot, and the lung was considerably inflamed. The *right* lobe was nearly obliterated, it had entirely lost its cellular appearance, and become dense, hard, and tenacious, and its size was not more than one eighth of that of the inflated lung. It appeared to us, that respiration had never been performed in that lobe from the time it received the wound. A small portion of serum was found in the pleura. The heart appeared to be considerably enlarged, and was irregular on its surface, at the places where it adhered to the pericardium. In cutting through its parietes, we found some parts of them nearly cartilaginous, and on opening the right ventricle, we were astonished to find three shot lying loose in its cavity. This ventricle was greatly enlarged, and lined with a thick coat, from which there projected numerous papillæ of a dun colour, giving it the appearance of the upper surface of the tongue of an ox. On opening the right auricle, we found two shot in its cavity, also lying detached. The internal surface of the auricle did not appear to have sustained much injury from their presence. The shot had entered the heart about one third of the way from its base to its apex, the wounds made by them were at a little distance from each other; they had all cicatrized, but the spots were plainly to be seen. In the cavity of the peritoneum, as in that of the pleura, there was a small quantity of effused serum. The liver appeared to be somewhat enlarged, but not otherwise much diseased, except about the gall bladder and its duct, where there were some grangrenous appearances, and part of the colon was also gangrenous.

In conclusion I may remark, that notwithstanding the entire loss of one half of the organ of respiration, and the presence in the other of a number of shot, the absorption of a part of the pericardium, the adhesion of another part to the mediastinum, and the presence of five shot in the cavities of the heart, this boy lived two months and six days.

The dissection was performed in the presence of a number of spectators of respectability, who were all eye-witnesses to the facts above stated.

LEONARD RANDAL, M. D.

*Purdy, Mc'Nary County, Ten. Sept. 15th, 1828.*

